# Alpha synuclein and inflammaging

**DOI:** 10.1016/j.heliyon.2025.e41981

**Published:** 2025-01-15

**Authors:** Geneviève L. Putnam, Robert W. Maitta

**Affiliations:** a University Hospitals Cleveland Medical Center, Cleveland, OH, USA; b Case Western Reserve University School of Medicine, Cleveland, OH, USA

**Keywords:** α-synuclein, Aging, Inflammaging, Inflammation, Parkinson's disease

## Abstract

The α-synuclein protein is an established molecule in Lewy body pathology, especially Parkinson's disease (PD). While the pathological role of α-synuclein (α-syn) in PD has been well described, novel evidence may suggest that α-syn interacts with inflammasomes in response to aging. As age is an inevitable physiological state and is also considered the greatest risk factor for PD, this calls for investigation into how α-syn, aging, and PD could be linked. There is a growing amount of data regarding α-syn normal function in the body that includes involvement in cellular transport such as protein complexes assembly, vesicular trafficking, neurotransmitter release, as well as immune cell maturation. Regarding abnormal α-syn, a number of autosomal dominant mutations have been identified as causes of familial PD, however, symptomatology may not become apparent until later in life due to compensatory mechanisms in the dopaminergic response. This potentially links age-related physiological changes not only as a risk factor for PD, but for the concept of “inflammaging ”. This is defined as chronic inflammation that accompanies aging observed in many neurodegenerative pathologies, that include α-syn's ability to form oligomers and toxic fibrils seen in PD. This oligomeric α-syn stimulates pro-inflammatory signals, which may worsen PD symptoms and propagate chronic inflammation. Thus, this review will explore a potential link between α-syn's role in the immune system, inflammaging, and PD.

## Highlights

• Alpha Synuclein has physiological roles important to organ systems outside the central nervous system.
• Alpha Synuclein interacts with elements mediating inflammatory processes.
• Alpha Synuclein may be important to inflammageing and changes associated with aging.
• Alpha Synuclein's role in immunity is indicative of the importance of this protein.

## 1 Introduction

Parkinson's disease (PD), a devastating and incurable illness, was first identified in 1817 by Dr. James Parkinson, who used the term “shaking palsy” to describe the syndrome's symptomatology [1]. This disease is a devastating neurodegenerative disorder affecting approximately 500,000 Americans with complications such as dystonia, impaired cognitive function, and dyskinesia [2,3]. Worldwide the disease affects as many as 6.1–8.5 million people with a lifetime incidence of approximately 2 %, thus representing one of the most debilitating neurological diseases [[4], [5], [6]]. Caused by the incremental loss of dopaminergic neurons in the substantia nigra, one of the primary suspects mediating PD pathology is α-synuclein (α-syn), a protein identified in 1994, whose sequence was found to be highly homologous to synucleins first described in the torpedo ray [7].

α-syn is a relatively small 140 amino acid long protein that is a major component of Lewy bodies (LB) found in PD [8,9]. It belongs to a protein family which also has two other members, β- and γ-syn, whose functions remain mostly to be determined [9]. Each synuclein has defined tissue distribution and apparently specific functions in the body, but α-syn is mostly found in the brain and ubiquitously minimally expressed in heart, lung, kidney and skeletal muscle [10,11]. The synuclein family shares a highly conserved sequence in vertebrates capable of oligomerization responsible for pathology [12], that for α-syn defines a disease group known as synucleinopathies [13]. α-syn is an intrinsically disordered and soluble protein monomer that can fold into a fibrillar conformation that promotes aggregation when mutated [14,15]. It is encoded by the *SNCA* gene, and mutations and multiplications of this gene are linked to familial forms of PD.

While the α-syn protein has been predominantly studied in neural tissues, it has also been found to exist in megakaryocytes and especially in platelets in high concentrations [16,17]. Interestingly, this protein has been shown to be essential for the maturation of lymphocytes and for normal hematopoiesis to occur [[18], [19], [20], [21]]. When α-syn is absent, marked disorders in lymphoid organ morphology, development and function of leukocytes, signs of anemia, platelet dysfunction, and lymphoid defects that include absent IgG production, cytokine dysregulation, and accumulation of intracellular inclusions among others are observed [21]. How α-syn mediates what appear to be multiple levels of function in normal hematopoiesis and lymphopoiesis remains to be elucidated. However, the domains of the protein that mediate these multiple functions in hematopoietic cells may be the same as observed in the central nervous system (CNS) [22].

α-syn is capable of bidirectional movement between the gut and the brain, and even though it is not known if this movement is part of its normal physiologic role or if this contributes to pathology, age may play a role in this mechanism [23]. Nevertheless, despite age being recognized as a risk factor in PD development, it is clear that age-related changes do not lead to every individual developing the disease. Humans accumulate age-related cellular damage; among them a systemic-long-term inflammation process described as “inflammaging” that may constitute the “first hit” in a series of events leading to development of age-related diseases [24]. Whereas a low-grade, chronic inflammatory state occurring in the CNS secondary to long-standing exposure to inflammatory stimuli, defines the hallmarks of the process known as “neuroinflammaging” which predisposes an individual to either cerebrovascular or neurodegenerative disorders [25]. Thus, an important area of interest is how inflammaging in peripheral systems could lead to cerebral PD pathology in select human populations. In this review, we will explore the basis by which α-syn may interact with inflammaging processes needed to be addressed in future experimentation, prevention, and treatment. To gather literature for this narrative review, PubMed was searched for articles using combinations of the following: α-synuclein, PD, inflammaging, neuroinflammaging, age, aging as search criteria.

## 2 Structure and function of α-synuclein

### 2.1 Wildtype and mutant structures

**Fig. 1:**
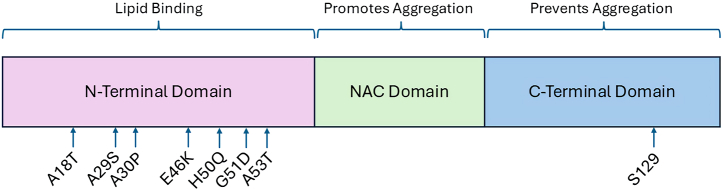
Representation of monomeric α-synuclein structure with most common point mutations in relation to the N-terminal domain. For reference serine residue 129 which undergoes phosphorylation in the C-terminal domain is shown. This figure is adapted from Refs. [165,166]. Graph shows the three known regions of α-syn. Amino terminus binds membrane structures, central domain is mostly freely accessible and carboxy terminus interacts with protein complexes.

The structure of α-syn spans three distinct domains. The first is the N-terminus, which is a positively charged, highly repetitive, conserved and amphipathic domain closely associated to or bound to lipid membranes (Fig. 1) [26]. In wild type (WT) α-syn, the first series of amino acids in this region prevent aggregation and induce α-helical formation at lipid membranes [27]. The core central hydrophobic (NAC) region is capable of forming fibrils and β-sheets. Lastly, the C-terminus is acidic, highly hydrophilic, and can take on the form of coils. Aggregation is also possible in this region if the random coil structure is electrostatically disrupted or if the serine 129 residue is dephosphorylated [28].

The first mutation identified was a G209A substitution corresponding to the A53T mutation. This mutation was found to be inherited in an autosomal dominant manner and was deemed responsible for early-onset PD [6,29]. Since then, other mutations identified were found to be associated with the familial form of the disease. On the other hand, sporadic cases account for the majority of PD patients. Of note, for the sporadic version of PD, the A18T and A29S mutations occur upstream of those that are familial [30]. Each of these mutations occurs in the amphipathic repeat region of the SNCA gene and can thus impact lipid interaction [14]. The protein forms two amphipathic helices that penetrate the lipid bilayer which becomes defective in the setting of mutations away from the N-terminal helix toward helix 2 possibly representing a step toward disease progression [31]. Nevertheless, the net effect of these point mutations is changes to lipid-induced fibril production that lead to pathology [32]. Since α-syn is the major component of Lewy bodies, mutations lead to changes in the rate of formation of these structures.

To date, a number of major *SNCA* point mutations have been described and have been linked to the familial form of the disease, including V15A, A30P, E46K, H50Q, G51D, A53V and A53T [[33], [34], [35], [36], [37]]. These mutations lead to distinct and at times dramatic phenotypes that for A53T, E46K, and H50Q result in promotion of higher rates of α-syn aggregation, A30P causes slower fibrillary formation, while G51D promotes cellular toxicity in the setting of reduced aggregation [38,39]. A53V mutations have strong family penetrance with more significant symptoms in families of a particular geographic region [40]. Experiments have shown that rapid formation of α-syn amyloid aggregates are seen with A30P, E46K, H50Q and A53T in liquid-liquid phase separation while A53E was characterized by reduced rate of aggregation [41]. G51D leads to earlier disease onset hinting to a distinct pathologic mechanism brought about by this mutation [39]. Since α-syn oligomers appear to be the neurotoxic form that causes cell death, mutations that facilitate their formation are likely more toxic [42]. However, even though the same mutation can be found in different individuals, there is great heterogeneity in the phenotype caused by them, as shown by E46K mutation which can range from limited aggressive disease to one that is more pronounced [43].

Based on aggregation results of α-syn mutations, nucleation-polymerization and nucleation-conversion polymerization processes can be reconciled into one continuous hypothesis [44]. In such hypothesis, A30P, E46K and A53T lead to polymorphic α-syn fibril formation in which the latter greatly enhances fibrils elongation in the presence of increasing α-syn seeding, thus explaining how this mutation results in early onset of familial PD [44]. This is partly due to A53T markedly enhancing α-syn nucleation rate through the conversion of monomers to amyloid nuclei and fibril elongation (similar to C-terminal truncation) [45]. Conversely, A30P can mediate faster oligomers formation with delayed conversion of these into fibrils [46]. For G51D, fibrils formed leads to a highly toxic form of α-syn [47,48]. On the other hand, E46K elicits a more pathogenic and stable fibril structure [49]. Mechanistically, A30P and A53T may also result in early disease onset as shown by patients expressing these mutations being devoid of brain vesicle-binding in the pre-synaptic region that profoundly disrupts neurotransmitter release [50]. Experimentally, neurons expressing A30P or A53T have reduced axonal transport [51]. Of interest, in animal models A30P, H50Q and G51D mutations result in reduced phosphorylation of serine 129 at the C-terminus, A53T leads to little change in phosphorylation, while E46K results in much higher phosphorylation [52]. However, it the presence of E46K mutation, phosphorylation of serine 129 may be rendered irreversible [52].

Recently, a new mutation (A30G) in a family of mediterranean origin, that leads to intrinsically disordered protein aggregates with reduced membrane binding and readily able to form fibrils was reported [53]. This mutation leads to a rapid conformational change with reduced helical structure in the portion of the protein embedded in the membrane bilayer resulting in inefficient anchoring and thus impaired vesicle docking at the synapse [54]. Separately, another α-syn mutation, A53E, results in a type of multiple system atrophy with Parkinson's-like phenotype suggesting that a mutation automatically does not quality as the cause of PD [55]. Taken together, all of these reports likely show that there is no unified mechanism mediated by α-syn mutations since each appears to affect function in specific ways.

Under homeostatic/physiologic conditions, α-syn takes on the form of a disordered monomer or helical tetramer that can perform neuroprotective inhibition of p53 (which can mediate cell cycle arrest, DNA repair, and apoptosis) and caspase-3 [56]. It has also been found to regulate synaptic vesicle release and trafficking [57]. When mutated, α-syn amyloid fibrils work as infectious agents and propagate in a prion-like manner that cause the formation and deposition of neurotoxic LB [15,42,56,57]. Nevertheless, even though the above-mentioned mutations have been being identified as potential causes of familial PD, gene multiplication, truncation, or overproduction are linked to sporadic cases, and the specific pathogenic structure in these instances remain unknown [56,58,59]. Interestingly, patients with sporadic disease may have greater amounts of α-syn in blood compared to those with familial (A53T) mutations suggesting that distinct deregulation of the protein's homeostasis occurs in sporadic vs. familial forms of the disease [60]. Furthermore, unlike the former, patients with this mutation can present with retinal changes that may represent a prodromal manifestation of disease [61].

### 2.2 Compensatory mechanisms in PD

PD is characterized by both motor and non-motor symptomatology. While motor functional changes do not become apparent until a greater depletion in dopamine occurs (>80 %) [62], some non-motor signs can manifest years earlier due to the chronic degeneration of dopaminergic neurons [63]. However, neuronal loss alone fails to explain interindividual differences in motor phenotypes and rates of functional decline [64]. This implies that alternate phenomena such as neuroplasticity in which nigral neuronal efficacy, changes in neurotransmitter conduction, expression of molecular mediators, and even increase in physical activity compensate for loss of dopamine and these play a major role during the earlier years of disease [65]. Favoring this view, neuroimaging patient data has shown that primary motor cortex-striatal networks, and specifically intercortical connections, become more efficient with progressive loss of dopaminergic terminals in the putamen suggesting that cortical plasticity in PD occurs [66]. Also, mutations in the glucocerebrosidase gene are characterized by early onset of symptoms, a more pronounced cognitive decline, and reduced levels of α-syn in cerebrospinal fluid [67], implying that this may constitute a compensatory mechanism. Animal data has also directly linked α-syn overexpression to mild losses of dopaminergic neurons and dopamine neurotransmitter levels in the substantia nigra, as well as residual dopaminergic neurons displaying smaller cell bodies, reduced dendritic branching and hyperexcitability [68]. Thus, taking abnormal α-syn propagation as a determinant of disease, data indicating improved parieto-premotor activity in patients with a milder motor phenotype favors a compensatory phenotype [64]. This indicates that nigral dopaminergic neurons are able to adapt during PD neurodegeneration. As a result, this level of neural adaptation represents an opportunity to develop new therapeutic agents to treat symptomatology.

### 2.3 Connections to PD

**Fig. 2:**
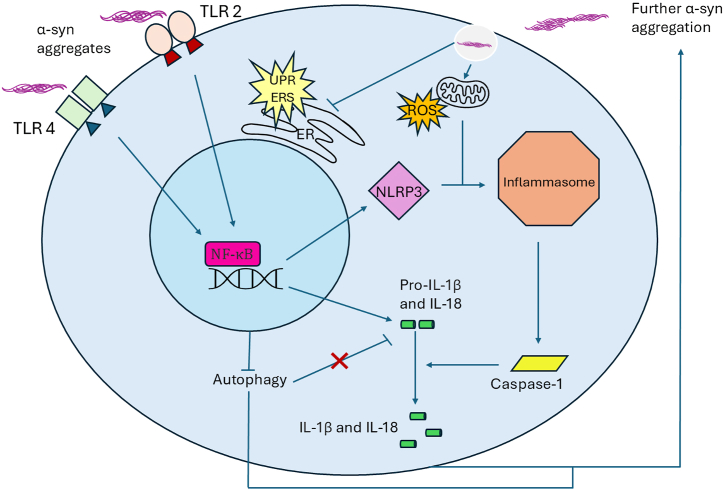
Diagram of α-syn interactions with inflammasome and inflammatory pathways in the cell. This includes activation of TLR-mediated pathways, gene expression, NLRP3 and the inflammasome, ROS and increase in inflammatory cytokines, activation of the unfolded protein response (UPR) and chronic endoplasmic reticulum (ER) stress (ERS) pathway, with feedback and communication among pathways that can be self-amplified with additional α-syn aggregation.

As mentioned earlier, the general consensus is that α-syn can act in a prion-like fashion and that elevated levels of aggregates or fragmentation of the protein accelerate LB formation [59,69]. Experimental data has shown that when mice are injected with only a single dose of α-syn fibrils, they develop LB capable of propagating throughout different cell types in neighboring brain regions [70]. Specifically, aggregation occurs in a sigmoidal fashion, where the initial lag phase accelerates into a growth phase as monomers preferentially build onto existing aggregates rather than other monomers. This aggregation can then promote formation of beta-sheet-rich amyloid fibrils that are capable of forming LB [57,71]. This is further supported by observations that when mutated, the “critical” N-terminal domain allows for enhanced binding while promoting oligomerization and LB formation [56]. Further studies have also found that truncated C-terminal domains can increase α-syn fibrillation [72,73]. The net result of aggregation is the inhibition of dopaminergic neurons leading to PD motor deficits [74,75]. Along these lines, overexpression of α-syn in an in vitro model has been reported to cause extensive dopaminergic neuronal loss after just three months [75]. This was further supported by studies showing that overexpression of WT α-syn in vivo and in vitro was detrimental to dopamine neurotransmitter transport due to the reduced concentration (40 %) of vesicles containing dopamine at synapses [76,77]. Accumulation of α-syn also leads to activation of the unfolded-protein response pathway in the endoplasmic reticulum (ER) in patients with PD [78], which when in high levels may create a more significant chronic ER stress accentuating neurodegeneration (Fig. 2) [79]. It is important to note that in sporadic cases of PD, α-syn levels are not overall increased but instead its phosphorylation or proportion of insoluble α-syn increases [80].

### 2.4 Global involvement of α-syn in the immune system

Although the role of α-syn in aggregate formation is important, this protein has also been linked to the activation of the innate immune response and maturation of the adaptive immune system [18,81,82]. Microglia, the myeloid-derived innate immune cells of the CNS, are responsible for phagocytosing infectious agents or native tissue debris caused by injury or cell death [80,83]. Once activated in this way, microglia undergo morphological changes, secrete cytokines or recognize CD4^+^ cells via major histocompatibility complex (MHC)-II antigen presentation [80,84]. However, there is still debate surrounding how microglia interact temporally with α-syn, since results have varied markedly depending upon the brain areas examined by a given study and the type of α-syn used in experiments [85]. For example, upregulation of MHC-II expression as well as genes for proinflammatory cytokines on microglia of PD patients has been reported [86]. Furthermore, this increase in microglial MHC-II expression has been observed in tandem with α-syn inclusions prior to neurodegeneration, suggesting that the initial buildup of α-syn caused the increase in MHC-II on microglia [80]. While this may not be the sole cause of neurodegeneration, the results allowed for a possible link between neuroinflammation followed by neurodegeneration to be formed [80]. Notably, microglia activation appears to occur prior to the accumulation of α-syn but the mechanism mediating this remains to be elucidated [84]. Nevertheless, even though these results were different from what was seen in microglia of the substantia nigra, the increase in cytokines after microglial activation was similar to previous studies supporting the conclusion that cytokines can drive dopaminergic neuronal dysfunction in PD patients [84,87]. Thus, it is apparent that α-syn accumulation is associated with increased MHC-II expression in immune cells [88].

It has been further proposed that α-syn aggregation may encourage phagocytic exhaustion, which can also lead to the degradation of dopaminergic neurons [89]. Regarding WT α-syn, microglia become activated during its release and promote autophagy to remove monomers via toll-like receptor 4 (TLR4) and the NF-κB-p62 inflammatory pathways before degeneration can occur (Fig. 2) [90,91]. In a similar manner, α-syn also interacts with TLR2 amplifying immune activation and chronic inflammation [91]. Importantly, many more proteins participate in this process, and thus mutations can lead to inappropriate autophagy (Fig. 2) [92,93]. Specifically, α-syn can activate TLR2 and TLR4 in a concentration-dependent manner and prevent proper microglial autophagy while regulating inflammation, thus contributing to neuroinflammation and PD pathology. It can also activate heterodimers of TLR2/TLR1 and TLR2/TLR6 resulting in increased production of tumor necrosis factor and interleukin (IL)-1β [94]. While this lends support to α-syn association with increases in p62 transcription (an autophagy receptor) that suggest suppression of autophagy, findings also support that α-syn could promote a non-transcriptional increase in p62 and prevent autophagy from taking place even when *de novo* synthesis is blocked [90,95].

MHC-II and α-syn are important to the adaptive immune response, since the former is responsible for antigen presentation to lymphocytes, especially T cells [95]. In α-syn-knockout (KO) mice, it was found that animals had markedly defective T cell maturation, as indicated by increases in CD4^−^CD8^−^ precursors and low mature CD4^+^ and CD8^+^ single positive T cells [18]. Importantly, remaining single positive CD4^+^ and CD8^+^ T cells were hyperactive as indicated by higher expression of early activation markers, increased IL-2 production and impaired Th2 differentiation [18]. Along these lines, absence of α-syn led to morphological changes in leukocytes indicative of intracellular transport deficits as indicated by accumulation of vacuoles and vesicles in cells [20]. This implies that α-syn aggregation can impact the homing abilities of helper T cells and microglial phagocytic functions [81,96,97]. These T cell deficits were accompanied by upregulation of IFN-γ, indicating enhanced pro-inflammatory activity of these cells capable of activating microglia [81]. Lastly, dopaminergic neurons in the substantia nigra expressed MHC-I when activated by cytokines released by microglia after α-syn exposure, and this MHC-I expression becomes a target for CD8^+^ T cells [98].

## 3 α-syn and SNAREs

### 3.1 SNARE structure and function

For neurological signaling to take place, neurotransmitters must be released via vesicular transport and travel across the synaptic cleft from a presynaptic neuron to bind to receptors on the corresponding postsynaptic neuron. Fusion of these synaptic vesicles to release neurotransmitters is dependent upon soluble N-ethylmaleimide-sensitive-factor attachment protein receptor (SNARE) complexes. SNAREs are highly conserved proteins and membrane fusion events include some of the better-known SNAREs including synaptobrevin2 (Syp2) [also known as vesicle-associated membrane protein 2 (VAMP2)], syntaxin-1 (Stx-1), and 25-kD synaptosome-associated protein (SNAP-25) [99]. However, it is important to note that there are 35 other known SNARE proteins and their use varies based on the type of synapse. SNAREs were originally divided into two broad groups: v-SNAREs (vesicle) and t-SNAREs (target), however, they may also be classified as r-SNARES (typically vesicular), or q-SNARES (usually on target membranes) since the t/v categorization could not account for alternative structures or homotypic events [21]. The SNARE proteins assemble into a highly stable coiled-coil tetramer called a synaptic fusion trans-complex to link the membranes of synaptic vesicles and presynaptic neurons for neurotransmitter release [6,100]. Typically, this requires one Stx-1, one VAMP2, and two SNAP-25 proteins. These trans-complexes transition to a cis-formation during fusion, which allows for dissociation due to ATPase activity via the recruitment of SNAP-25 and thus permitting SNARE proteins to be recycled for future synaptic fusion events [6,[101], [102], [103], [104]].

### 3.2 SNAREs and immunological synapse

There is general agreement based on in vivo and in vitro studies that α-syn promotes SNARE docking and complex assembly in presynaptic neurons via the cross-linking of its C-terminal region with the N-terminal domain of VAMP2 [[105], [106], [107]]. Experiments have shown that using high levels of exogenous α-syn inhibits vesicular fusion to the synaptic membrane, which confirms that the protein regulates SNARE function [108,109]. SNAREs also play a key role in the transport of cell signaling molecules, which are vital for α-syn to carry out its interactions with the immune system. This is especially the case for phagocytic cells such as microglia and mechanisms mediating autophagy. Data indicates that when microbial invasion occurs, Stx11 and SNAP-23 (the latter of which is located in macrophages) work together to transport TLR4 to allow for secretion of pro-inflammatory factors [110,111]. Stx11 has also been reported to be upregulated on monocytes and compete with other SNAREs to downregulate clearance of apoptotic cells by macrophages [112]. Likewise, SNAP-23 has been found to mediate maturation of phagosomes and FcR-mediated phagocytosis in macrophages [113]. Similarly, Stx17 localizes to autophagosomes for targeting the endosomes/lysosomes for degradation [114]. Cumulatively, this data indicates that SNARE complexes are essential for normal immune responses to be carried out throughout the body.

In addition to the role of SNAREs in the innate immune system, they have also been shown to influence T cell infiltration. MHCs on antigen-presenting cells present antigens to T cell receptors (TCRs) resulting in T cell activation [115]. This immunological synapse between the two cell populations is a hub for vesicle trafficking that drives the secretion of cytokines and other signaling molecules as part of the adaptive immune response [116]. Regarding SNARE involvement, results have shown that after TCR activation, a membrane-bound molecule called the linker for activation of T cells is transported to the Golgi in a retrograde manner by Stx16, while VAMP7 assists this linker's anterograde transport to the immunological synapse [117]. An additional pathway post-TCR activation involves VAMP8 and Stx11 interaction with SNAP-23 and Stx4 to exocytose recycling endosomes. This is of importance in T cell cytotoxic activity via granular release that is mediated by Stx11/SNAP-23 complexes [118,119]. Syp2 is also implicated in granule-specific release in a way similar to that in neuronal axons [120]. Additional SNARE proteins known to be involved in T cell cytotoxicity include Stx8 for lytic granule trafficking while SNAP-23, Stx3, and Stx4 mediate chemokine release [121,122].

### 3.3 SNARE interaction with α-syn under homeostatic conditions

It should be evident that even though α-syn has been shown not only to interact but influence SNARE complex assembly, the exact physiological underpinnings of this interaction remain to be defined [123]. In vitro findings have shown that inhibition of SNARE complex formation is dependent upon the concentration of α-syn and its interaction with the membrane bilayer (not the complex directly), so long as there are accessible lipids [108]. In particular, high concentrations of monomeric α-syn inhibits vesicle docking via the binding of acidic lipids, but does not come into contact with VAMP2 [82]. Arachidonic acid stimulates SNARE complex assembly; however, experimental data indicates that α-syn sequesters arachidonic acid and thus prevents SNARE activation for fatty acid exocytosis regardless of acid concentration [124]. Notably, this is not the case in the absence of arachidonic acid regardless of α-syn concentration, suggesting that arachidonic acid acts as a modulator of SNARE complexes while α-syn exerts its effect on SNARE complexes without direct interaction with SNARE proteins [124].

Conversely, other studies have suggested that α-syn promotes SNARE docking and therefore complex assembly via the cross-linking of its C-terminal region with the N-terminal domain of VAMP2 [105]. This can occur in a nonclassical chaperone mechanism in which α-syn directly supports SNARE complex formation through a binding pattern similar to that seen in neurons [123]. Still, others have suggested that this occurs via α-syn clustering of vesicles in active zones to increase complex assembly kinetics that are dependent on the presence of anionic lipids [125]. SNAREs themselves have also been shown to control α-syn release in a calcium-dependent manner that is regulated by the autophagy-lysosomal pathway [126]. The t-SNAREs involved in this process were Stx4, SNAP-23, and VAMP3/7/8 all of which played a role in α-syn exocytosis, although VAMP8 played the largest role [126].

### 3.4 SNARE interaction with α-syn mutants

Analysis of the transcriptomes of PD patients indicated that the main effect of mutations was over exocytosis and SNARE interaction, including VAMP2 and Stx1 [127]. When VAMP2 interacts with oligomers of α-syn, it can no longer bind to t-SNAREs, preventing exocytosis [127]. Of the known mutations identified as both familial and capable of inhibiting fusion, E46K did so more than WT α-syn, A30P was less capable, while A53T was shown to have equal inhibitory effects as WT [27,82,108]. A53T has also been linked to lower ER/Golgi SNARE assembly in vitro that can be rescued by overexpression of SNAREs to replace those that are inactivated [128,129]. Of note, if phosphatidylserine is removed from t-SNARE vesicles α-syn can prevent docking, and if the α-syn C-terminus that binds VAMP2 is shortened docking is completely inhibited [105].

Lastly, large oligomers of α-syn preferentially bind multiple VAMP2 proteins, preventing vesicle docking via t-SNAREs and even when α-syn was overexpressed forming only soluble aggregates, SNARE function was impeded [130]. This is further supported by a general increase in α-syn oligomers and decrease in SNARE proteins in the cerebrospinal fluid of PD patients [131]. However, in the presence of α-syn mutations disruptions of SNARE complex docking or assembly capable of impacting the immune system and specifically immune cell signaling can occur [21].

## 4 Inflammaging and PD

### 4.1 Inflammasome structure and activation

As previously discussed, the immune response in PD is mediated for the most part by microglia, which secrete cytokines once activated that elicit neuroinflammation. In response to such signaling, a multiprotein complex called an inflammasome is activated which results in secretion of pro-inflammatory factors and activation of the innate immune system [132]. Specifically, there are two signals needed for this activation, the first being detection of a damage-associated molecular pattern by a pattern recognition receptor (PRR). The second is the upregulation of necessary genes such as pro-IL-1β and NOD-like receptor protein 3 (NLRP3) a type of cytoplasmic PRR mainly studied in the context of neurodegenerative diseases, age-related animal models, and in the elderly [[132], [133], [134]]. This second signal is responsible for the oligomerization of NLRP3 to promote activation of pro-caspase-1 (Cas-1), which cleaves IL-1β and IL-18 precursors to generate their active forms [135,136]. Together with the TLRs, this defines the α-syn/TLRs/NLRP3/Cas-1 inflammasome axis mediating pro-inflammatory pathways seen in PD [85,137].

### 4.2 Inflammaging and its causes

Inflammaging is a result of an individual's exposure to external and internal stresses that trigger inflammation via the immune system. As we age, this can result in a low-grade, chronic, systemic pro-inflammatory state which has been termed “inflammaging” [138]. One possible mechanism is through the accumulation of senescent cells as we age, that results in a senescence-associated secretory phenotype made up of pro-inflammatory cytokines such as IL-6 that contribute to inflammaging [139,140]. An additional mediator is oxidative stress and production of reactive oxygen species (ROS), which increase as we age and promotes senescence and cell death while also supporting an oxidative state and inflammasome assembly [141,142]. ROS is a byproduct of mitochondrial aerobic respiration that can impair ATP production. This is supported by reports showing that older organisms have fewer and less efficient mitochondria that produce more ROS compared to younger organisms [143,144]. On the other hand, senescence and ROS production can be promoted by inflammaging itself, creating a self-propagating loop that is central to understanding the human aging process [141].

### 4.3 Inflammaging in the brain/neuroinflammaging

Unfortunately, inflammaging can spread systemically and therefore impact the whole organism, including the brain. NLRP3 in the inflammasome may steer inflammaging and primarily cause microglia to produce inflammation-inducing mediators [145]. This is suspected because aged mice have elevated levels of the inflammasome machinery, especially NLRP3 in microglia [146]. This is supported by the acquired protection from inflammaging and improved cognitive performance after NLRP3 inflammasome removal [136,147]. As microglial senescence develops with increasing age, there is a corresponding increase in neuroinflammatory factors such as IL-1β, IL-6, and ROS that “prime” microglia and drive neurodegeneration [148]. Moreover, when the inflammasome is overactivated it accelerates inflammaging and neurodegeneration in various disease states. For example, aged humans with high levels of inflammasomes experience hypertension, arterial stenosis and overall shorter lifespans [149]. Of note, murine models have indicated that age follows a propagation pattern, similar to α-syn in PD. When blood from aged animals was transfused into younger mice, it resulted in decreased synaptic plasticity and memory, while the reverse led to aged mice being rescued from cognitive impairment [150,151]. Lastly, dopaminergic neurons exhibit increased vulnerability to oxidative stress that becomes more pronounced in aging PD subjects [152].

### 4.4 Inflammaging and α-syn

Although there is an association between biological aging and inflammation, not everyone develops PD despite age being a major risk factor. Therefore, it would be beneficial to analyze how α-syn is affected by inflammaging to gain a better understanding of processes that lead to PD. In young mice, microglia phagocytose both free and exosome-bound α-syn more efficiently compared to older mice (Table 1) [153]. A marked difference between the two groups is that older mice secrete higher levels of cytokines from microglia indicative of a hyperactivated state [153]. The inhibition of exocytosis by α-syn has direct links to SNAREs since soluble monomeric α-syn is still needed for normal SNARE complex assembly and membrane fusion in aging mice. When dopaminergic neurons undergo age-induced oxidative stress, α-syn fibrils promote aggregation and the microglial NLRP3 inflammasome is activated by either of these triggers [154]. Interestingly, inhibition of the inflammasome ameliorates PD symptoms, suggesting that the NLRP3 inflammasome may be a source of inflammaging and corresponding PD pathology [154]. This aggregation would be made worse during aging due to decreases in pH, which stabilize α-syn dimers [155].

**Table 1:** Studies supporting association of α-syn and inflammaging.

Reference	Main Findings	
[87]	Early microgliosis associated with α-syn accumulation and neuronal dysfunction.	
[86]	Presence of α-syn: 1) Detrimental to macroautophagy and protein synthesis pathways; 2) Changed immune response and axonal degeneration pathways; 3) Upregulated MHC-II receptor expression and genes for proinflammatory cytokines; 4) Indicated endocytosis, inflammation and axonal function are compromised in early PD stages	
[80]	Increase in microglial MHC-II expression in tandem with α-syn inclusions prior to neurodegeneration.	
[90]	1) WT α-syn activated microglia and promoted autophagy to remove monomers via TLR4; 2) Disruption promoted accumulation of misfolded α-syn resulting in midbrain dopaminergic neuron degeneration.	
[95]	1) Extracellular WT human α‐syn activated TLR4, resulting in inflammatory responses; 2) Autophagy suppressed in microglia via TLR4‐dependent p38 and Akt/m TOR signaling cascades that aggravated α‐syn‐induced inflammatory responses.	
[153]	1) Compared to old mice, microglia phagocytosed both free and exosome-bound α-syn more efficiently in young mice; 2) Older mice secreted higher levels of cytokines from microglia, consistent with hyperactivated state.	
[154]	1) Both NLRP3 and ASC localized to hypertrophic reactive microglia in PD patients; 2) The kinetics and activation profile of NLRP3 induced by α-syn fibrils was distinguishable from conventional activators; 3) Release of active IL-1β p17, cas-1 p20 and ASC in absence of pyroptosis was unique to NLRP3 activation with α-syn fibrils.	
[138]	1) Rotenone induced α-syn accumulation in both young and aged mouse groups, yet only the latter exhibited neurodegenerative changes with motor impairment; 2) Inflammaging was observed in the OB and striatum of 12-month-old mice compared to 3-month-old mice; 3) Age deemed a critical factor for α-syn induced neuroinflammation and neurodegeneration; 4) Low-dose exposure to rotenone to mimic aging in young mice induced LB inclusions in the brain; 5) Inferred age is pivotal factor in α-syn-induced neuroinflammation and neurodegeneration.	
[158]	1) The C/EBPβ/AEP inflammatory pathway age-dependently activated and cleaved α-syn N103 residue to regulate Lewy body-like spread in human α-syn transgenic mice; 2) Deletion of C/EBPβ or AEP substantially diminished oxidative stress, neuro-inflammation, and PD pathology.	
[159]	Human α-syn mice that exhibited chronic neuron-specific oxidative stress had significantly higher α-syn aggregate densities than littermate mice.	
[160]	Exogenous α-syn inhibited IL6ST, which in turn inhibited JAK2-STAT3 pathway in microglia while increasing HIF-1α thus accelerating oxidative stress.	
[145]	1) α-syn activated the NLRP3 inflammasome through microglial endocytosis and subsequent lysosomal cathepsin B release; 2) Deficiency of cas-1, part of the NLRP3 inflammasome, significantly inhibited α-syn-induced microglia activation and IL-1β production.	

Key to table abbreviations: ASC = apoptosis-associated speck-like protein containing a C-terminal caspase recruitment domain; TOR = target of rapamycin; IL6ST = interleukin 6 cytokine family signal transducer; HIF1α = hypoxia inducible factor 1 subunit alpha; OB = olfactory bulb; AEP = asparagine endopeptidase; cas-1 = caspase-1; PD= Parkinson's disease; TLR4 = toll-like receptor 4; NLRP3= NOD-like receptor protein 3; WT = wild type.

Additional mechanisms of age-related α-syn aggregation due to oxidative stress during aging may include direct modification of α-syn such as nitrosylation, truncation, and dimerization, or indirect methods such as autophagy impairment and DNA damage [156]. Unfortunately, experimental models of inflammaging in the brain are not well established. However, EAAC1 KO aged mice used to model chronic oxidative stress were characterized by activated c-Abl, a DNA damage response kinase located in the cytosol, which promoted phosphorylation of α-syn resulting in slower autophagy rates [156]. Overexpression of α-syn also increased aggregation in aged mice [156]. Importantly, a murine hypoxia model indicated that induction of α-syn phosphorylation triggered higher proportion of aggregation and propagation compared the non-phosphorylated form of the protein that resulted in dopaminergic neuronal loss similar to that seen in PD [157].

Chemical compounds such as rotenone can simulate inflammaging as that seen in PD but without dopaminergic neuronal loss. In mice, rotenone induced α-syn accumulation in both young and aged mouse groups, yet only the latter exhibited neurodegenerative changes with motor impairment [138]. One of these pathways regulating inflammation in response to glial activation is the C/EBPβ/AEP pathway induced by rotenone, thus linking it to the active aggregation and cleavage of α-syn [158]. Of note, aggregation itself can promote inflammaging as shown in mice [159]. Microglia-derived mediators also promote the hyperactivity of glial cells via the STAT3 pathway which can upregulate transcription of genes responsive to hypoxic conditions and cytotoxic damage [160]. This pathway can be inhibited by exogenous α-syn through reduction of oxidative stress repair (increasing ROS) by inhibiting IL-6 and STAT3 in microglia (Table 1) [160].

### 4.5 Conserved structures and repetitive cycles

Throughout the cell types, proteins, and complexes that have been reviewed, there is a unifying theme of conserved structure and shared function. It is apparent that α-syn structural integrity is an important factor that requires further investigation [161]. Likewise, SNAREs are highly conserved and have shown essential to both normal brain physiology and potentially immune responses. This favors the use of animal models to study α-syn since the roles thus far shown to be mediated by this molecule are likely conserved between species. There are feed-forward mechanisms that can be pathological such as the prion-like propagation of α-syn. In this regard, when oligomerization begins, the protein preferentially adds to existing aggregates and drives sigmoidal growth [57]. This emphasizes the importance of understanding how a protein necessary in normal cell functioning can lead to disease due to structural or conformational changes. At the cellular level, primed or hyperactive microglia can also set in motion α-syn aggregation which causes inflammation and feeds back further activation of primed microglia [148]. The latter also carry out autophagy, but if α-syn oligomers bound to VAMP2 prevent SNARE complex assembly, the aggregates will not be cleared [21]. Simultaneously, if dopaminergic neurons needed to inhibit NLRP3 activation are degraded, oligomerization can be exacerbated, leading to enhanced neuronal degradation [148].

Consequently, taking these looping mechanisms into account, it is feasible that since aged organisms generally have elevated levels of α-syn, there is a predisposition to greater numbers of inflammasomes (specifically NLRP3 in microglia) in aged subjects [146,162]. To draw a connection of how inflammaging can lead to excessive inflammasome activation, it has been suggested that inflammaging is capable of stimulating the lymphatic system through impairment of the blood-brain barrier secondary to ROS-mediated injury allowing entry of inflammatory mediators into the CNS [136]. Once inside the brain, microglial NLRP3 inflammasome activation increases α-syn aggregation and expands ROS synthesis stimulating α-syn aggregation via oxidation [136]. The fibrillar form of α-syn can, in turn, impair proper mitochondrial function and cause further ROS production, thus driving a higher inflammatory microglial state [144]. As mentioned earlier, α-syn aggregation can inhibit SNARE complex assembly and access to vesicles, which can result in dysfunction of the immune system (including microglial autophagy), signaling pathways and expand further the inflammatory cycle [163]. Dopaminergic neurons may have greater susceptibility to oxidation and consequently degradation, events that drive PD progression through promotion of inflammation [144,159].

### 4.6 Limitations and future directions

Despite a number of technical limitations, researchers should be able to study though the induction of chronic oxidative stress, how this process mimics inflammaging while evaluating its effects on α-syn aggregation. Mimicking inflammaging would ideally be a drawn-out process, due to the requirement of simulating an organism's natural aging process. For example, low-dose exposure to rotenone in young mice induces LB inclusions in the brain [138]. However, this would not be practical to do during the life of an animal to mimic aging progression. The use of chemicals introduces another variable difficult to control for, which is the extent that off-target effects impact other organ systems. These are just a few of the potential challenges encountered developing proper experimental models to study these interactions.

One possibility may be the use of brain organoids to model PD progression in human tissue, especially in light of advances in regenerative medicine that may allow for portions of the brain involved in PD to be analyzed [164]. Investigation into the inflammatory state and α-syn profile of the elderly could yield more information on why some older humans develop PD while others do not. Results from such studies could then be used to identify biomarkers that increase the propensity for PD diagnosis or development of symptomatology. Studies of age-related treatments would also be possible since blood donations from young mice has been able to rescue cognitive function in aged mice [151]. These findings ought to stimulate the interest of researchers to explore how this can give origins to new treatments for PD patients.

In summary, α-syn represents an important protein that not only leads to PD pathology but that during normal physiology mediates important processes not limited to the CNS. Indeed, its role as an important mediator in the maturation of immune cells, interaction with SNARE complexes but above all inflammatory mediators, make this protein's study essential to establish not only a more complete knowledge of its function but potentially develop targeted therapeutic approaches that lead to not only improvement of PD function but addresses chronic inflammatory mechanisms playing out during aging.

## CRediT authorship contribution statement

**Geneviève L. Putnam:** Writing – original draft, Investigation, Formal analysis, Data curation. **Robert W. Maitta:** Writing – review & editing, Writing – original draft, Supervision, Methodology, Investigation, Formal analysis, Data curation, Conceptualization.

## Declaration of competing interest

The authors declare the following financial interests/personal relationships which may be considered as potential competing interests:Robert Maitta is Associate Editor for Heliyon. All authors declare that they have no known competing financial interests or personal relationships that could have appeared to influence the work reported in this paper.
